# Exploiting Online Spatially Resolved Dynamic Light Scattering and Flow-NMR for Automated Size Targeting of PISA-Synthesized Block Copolymer Nanoparticles

**DOI:** 10.1021/acspolymersau.4c00074

**Published:** 2024-11-26

**Authors:** Peter M. Pittaway, Kudakwashe E. Chingono, Stephen T. Knox, Elaine Martin, Richard A. Bourne, Olivier J. Cayre, Nikil Kapur, Jonathan Booth, Robin Capomaccio, Nicholas Pedge, Nicholas J. Warren

**Affiliations:** a School of Chemical and Process Engineering, University of Leeds, Woodhouse Lane, Leeds LS2 9JT, U.K.; b School of Mechanical Engineering, University of Leeds, Woodhouse Lane, Leeds LS2 9JT, U.K.; c Pharmaceutical Technology & Development, Operations, AstraZeneca, Silk Road Business Park, Macclesfield SK10 2NA, U.K.

**Keywords:** digital chemistry, polymerization, online analysis, nanomaterials, autonomous manufacturing, flow chemistry

## Abstract

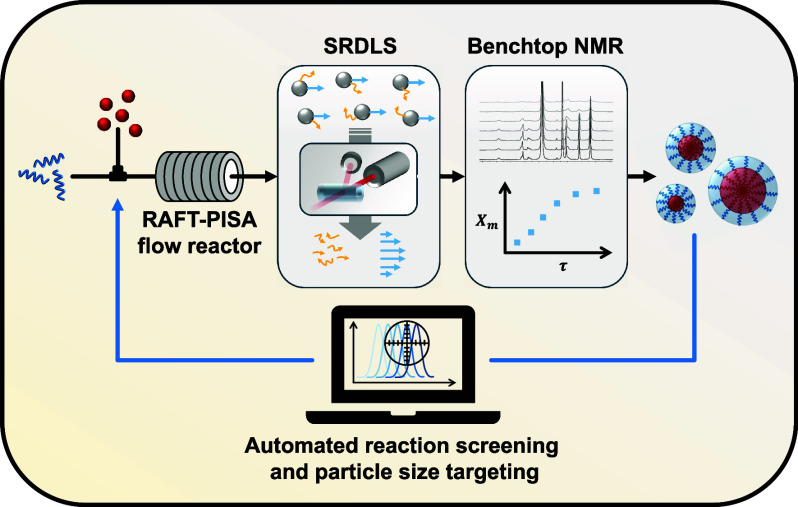

Programmable synthesis of polymer nanoparticles prepared by polymerization-induced self-assembly (PISA) mediated by reversible addition–fragmentation chain-transfer (RAFT) dispersion polymerization with specified diameter is achieved in an automated flow-reactor platform. Real-time particle size and monomer conversion is obtained via inline spatially resolved dynamic light scattering (SRDLS) and benchtop nuclear magnetic resonance (NMR) instrumentation. An initial training experiment generated a relationship between copolymer block length and particle size for the synthesis of poly(*N*,*N*-dimethylacrylamide)-*b*-poly(diacetone acrylamide) (PDMAm-*b*-PDAAm) nanoparticles. The training data was used to target the product compositions required for synthesis of nanoparticles with defined diameters of 50, 60, 70, and 80 nm, while inline NMR spectroscopy enabled rapid acquisition of kinetic data to support their scale-up. NMR and SRDLS were used during the continuous manufacture of the targeted products to monitor product consistency while an automated sampling system collected practically useful quantities of the targeted products, thus outlining the potential of the platform as a tool for discovery, development, and manufacture of polymeric nanoparticles.

## 1 Introduction

New approaches to the lab-scale discovery of high-value chemical products have emerged, which employ the benefits of continuous-flow processing, online analysis, and automation.^1−6^ Synergistically, these developments led to reactor platforms capable of synthesizing, analyzing, and optimizing chemical reactions with little existing knowledge of the system.^2^ The discovery and development of next-generation functional polymer products stands to benefit from the application of these “smart” reactor platforms given the vast chemical and process parameter spaces to be explored. Flow chemistry is becoming a popular tool for polymer chemists since flow systems are easily automated and scaled and give rise to precisely controlled and reproducible reaction conditions.^3,7,8^ Closing the loop between flow synthesis and autonomous discovery requires the application of inline and online monitoring techniques to enable real-time product characterization. For polymer synthesis, numerous techniques have been reported,^9,10^ with some already incorporated within self-optimizing platforms.^11,12^ For example, online conversion monitoring has been achieved using spectroscopic approaches including UV–visible,^13,14^ IR,^15−18^ and Raman.^19,20^ More recently, inline benchtop NMR spectroscopy has become a routine technique for elucidating monomer conversion.^11,21−23^ For systems that undergo particle formation, particle size and particle size distributions (PSD) are key properties governing product performance^24^; however, at present, there are few methods available for collecting online particle size data. Turbidimetry has been suggested, though is only quantitatively useful in very dilute samples, requiring assumptions to be made about the shape of the PSD.^25−27^ Small-angle X-ray scattering (SAXS) has been used to perform in situ studies of particle size and morphology during the growth of block copolymer nanoparticles in batch^28,29^ and flow reactors^30^; however, high cost and limited access to X-ray facilities can make the technique prohibitive. On the other hand, the use of dynamic light scattering (DLS) is widespread for postsynthesis characterization but requires samples to be both static and dilute. Characterization of particle size as a hydrodynamic diameter in concentrated dispersions has been demonstrated using low-coherence DLS^31^ and further decoupled from convective motion using optical coherence tomography.^32^ The low coherence interferometric setup of spatially resolved DLS (SRDLS) allows capture and processing of light scattered from localized regions within a sample volume, allowing a spatial “map” of particle sizes and distribution in contrast to conventional DLS which measures scattered light from a single larger sample volume.^32,33^ Based on these principles, spatially resolved DLS (SRDLS) has emerged as a novel process analytical technology (PAT) tool to monitor particle size in concentrated, flowing suspensions,^33^ offering the potential for application in continuous-flow synthesis platforms, which is yet to be exploited.

One class of promising nanomaterials, which would benefit from an ability to accelerate development, are block copolymer nanoparticles, which have applications in areas including energy,^34−36^ theranostics,^37−40^ nanofabrication,^41,42^ advanced materials,^43−45^ and drug delivery.^46,47^ An efficient method of preparing these materials is via polymerization-induced self-assembly (PISA), which can be facilitated by reversible addition–fragmentation chain-transfer (RAFT) mediated aqueous dispersion polymerization.^48^ A well-reported aqueous PISA formulation comprises a soluble poly(dimethylacrylamide) (PDMAm) macromolecular chain transfer agent (macro-CTA), which is chain-extended with a water-soluble monomer diacetone acrylamide (DAAm).^30,49−52^ With such a synthesis, the PDAAm block reaches a critical degree of polymerization (DP) where it becomes hydrophobic resulting in amphiphilic polymer chains. These PDMAm_*x*_-*b*-PDAAm_*y*_ block copolymer chains undergo spontaneous self-assembly to form nano-objects comprising a PDAAm core and a PDMAm “stabilizer” block. By manipulating hydrophilic PDMAm and hydrophobic PDAAm block lengths as well as product concentration, it is possible to tailor the size and morphology of these particles in batch polymerization.^50^ Recently, it has also been shown that this process can be facilitated in a flow reactor, whereby the flow rates, temperatures, residence times, and reactor geometries can all be manipulated to modulate properties to the same effect.^49,51,52^ Furthermore, it has been shown that benchtop NMR is a convenient method of monitoring the kinetics of this process in real time.^23^ Although monitoring kinetics is important, the most useful characteristic to measure in this case is the particle size and morphology, which in the aforementioned studies required manual and time-consuming postprocess analysis. Achieving this in real time will accelerate the discovery and development of these materials. While online size measurements in these systems have been possible in flow using SAXS, this is a highly specialized technique with limited accessibility.^30^

Herein, we report a continuous-flow reactor platform with inline SRDLS and apply it for the continuous determination of nanoparticle hydrodynamic diameter, referred to as particle size during RAFT dispersion polymerization. The automated system coupled with rapid data acquisition afforded by inline SRDLS enables particle sizes to be rapidly targeted, while integration of inline NMR spectroscopy offers additional characterization and convenient collection of reaction kinetic data. Finally, production of these targeted products is scaled by continuous manufacture to obtain larger volumes of samples using an automated sampling system.

## 2 Experimental Section

### Materials

*N*,*N*-Dimethylacrylamide (DMAm; 99%, 500 ppm MEHQ) and 4,4′-Azobis(4-cyanovaleric acid) (ACVA; ≥ 98%) were purchased from Sigma-Aldrich (UK) and used as received. 3-((((1-Carboxyethyl)thio)carbonothioyl)thio)propanoic acid (CCTP) was purchased from Boron Molecular (USA) and used as received. Diacetone acrylamide (DAAm) (Alfa Aesar, 99%) was used as received. The poly(dimethylacrylamide)_150_ (PDMAm_150_) macro-CTA chain-transfer agent (macro-CTA) was prepared according to the procedure below to use as the chain-transfer agent, and 2,2′-azobis[2-(2-imidazolin-2-yl)propane]dihydrochloride (VA-044) (Fujifilm Wako Chemicals, ≥ 97%) as the initiator in pH 2.5 water was used.

### Batch Synthesis of Poly(dimethylacrylamide)_150_ (PDMAm_150_) Macro-CTA

PDMAm_150_ was prepared in a batch and purified according to the following procedure for use in subsequent flow experiments. DMAm (50.67 g, 0.511 mol, 150 equiv), CCTP (0.87 g, 3.42 mmol, 1 equiv), and 1,4-dioxane (110.46 g) were added to a round-bottomed flask containing a PTFE stirrer bar. To a separate vial, ACVA (0.095 g, 0.34 mmol, 10 equiv) and 1,4-dioxane (10.46 g) were added. The round-bottomed flask was submerged in a temperature-controlled (75 °C) oil bath and stirred at 300 rpm while both containers were sparged with nitrogen for at least 30 min. A syringe was then degassed and used to transfer the ACVA solution into the round-bottomed flask to give a 30% w/w reaction solution. After 85 min, the flask was removed from the oil bath and quenched by exposing it to oxygen in the air while cooling under cold water. Once cooled, the reaction mixture was precipitated from a dropping funnel into a rapidly mixed excess of diethyl ether. The precipitate was then filtered, washed, and dried overnight.

### Reactor Platform

A tubular perfluoroalkoxy alkane (PFA) reactor (8.5 mL, 1/8 in. OD, 1.59 mm ID) was heated by submerging in a temperature controlled oil bath (set to 75 °C). Reagents were delivered by using a system of four pumps in addition to a separate pump for a MeOH and water mixture (80:20 by volume) for cleaning between reactions. A Chemyx Fusion 4000X syringe pump was used to deliver separate feeds of macro-CTA solution; a pair of Teledyne ReaXus 6010R HPLC pumps supplied two separate feeds of the monomer solution, and an additional HPLC pump was used for the wash mixture. An SRDLS instrument (InProcess-LSP) fitted with a micro flow cell (1 mL internal volume) was installed after the reactor, and the outlet of the SRDLS instrument was passed directly through a low-field (60 MHz) benchtop NMR spectrometer (Magritek). Beyond the NMR, the flow was directed by an 8-way selection valve to either a 1 L waste bottle or to one of seven 100 mL sample bottles. Compressed air was used to pressurize these outlet containers to 1.5 bar as a means of providing back-pressure to the reactor. To evaluate monomer conversion in the present system, it was necessary to collect spectra of the unconverted reaction mixture. This was conveniently achieved by installing a 6-way switching valve immediately before the reactor to divert the reaction mixture to the NMR prior to each reaction.

**Figure 1:**
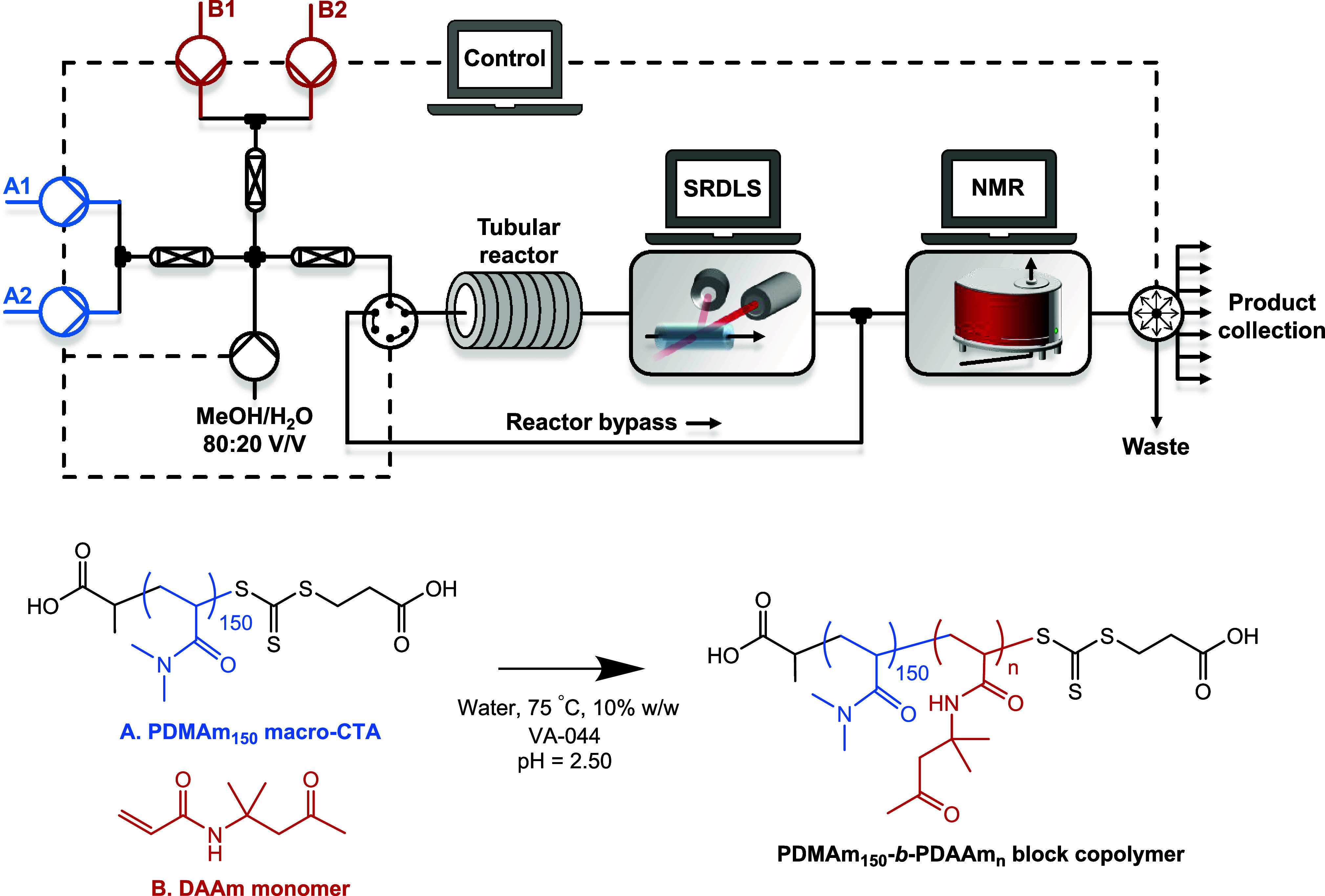
(Top) Schematic of the reactor platform for synthesis and inline characterization of PDMAm-*b*-PDAAm block copolymer nanoparticles. (Bottom) Reaction scheme for the aqueous dispersion RAFT polymerization of DAAm using a PDMAm_150_ macromolecular chain-transfer agent.

Four flasks were prepared to perform each series of reactions: two (A1 and A2) containing solutions of PDMAm_150_ macro-CTA and VA-044 in pH 2.5 water and two (B1 and B2) containing solutions of DAAm in pH 2.5 water. A set of material balances (Equations S1–S8) were constructed for this four-pump configuration such that copolymer degrees of polymerization (DPs) could be targeted by adjusting the relative ratios of pumps A1 to A2 and B1 to B2. The minimum DP was achieved when only pumps A1 and B1 were running and the maximum when only pumps A2 and B2 were running. Both flasks containing macro-CTA solution had the same CTA to initiator ratio but in different total concentrations (% w/w), and similarly, both monomer flasks had different concentrations. In this way, DPs could be targeted between the limits of the minimum and maximum with a fixed CTA to initiator ratio and final product concentration. After each T-junction, packed bed mixers containing glass beads were used to ensure reagents were well mixed before reaching the reactor.^53^ A full schematic of the reactor platform is shown in Figure 1 and a photograph is shown in Figure S1.

### Continuous-Flow Synthesis of Poly(dimethylacrylamide)_150_-*b*-poly(diacetone acrylamide)_*n*_ (PDMAm_150_-*b*-PDAAm_*n*_)

A general procedure for performing flow reactions involved the following. Two pairs of flasks were prepared with different concentrations to span DPs ranging from 50 to 650. For this range, the flask concentrations (% w/w) were calculated from Equations S1–S4; A1 = 2.43%, A2 = 12.88%, B1 = 17.58%, and B2 = 7.18%, to yield a final product concentration of 10%w/w. Flasks A1 and A2 contained macro-CTA and initiator in a 5:1 molar ratio, respectively. Once prepared, flasks A1 and A2 were sparged with nitrogen for at least 30 min before being loaded into gastight stainless steel syringes and fitted to the syringe pump. Flasks B1 and B2 were not sparged since this was found to result in the gradual autopolymerization of DAAm within the stock solution. For all reactions, the oil bath containing the reactor was maintained at 75 °C. After loading the flasks, the system was prepared by flowing at least 5 reactor volumes of the MeOH/water mixture through to remove any trapped air bubbles while the compressed air was opened to the collection bottles to pressurize the system. The experiment was then started from the Python interface while monitoring of particle size and conversion commenced using the SRDLS and NMR.

Three continuous-flow experimental routines were performed. The first involved automated screening of PDMAm_150_-*b*-PDAAm_*n*_ block copolymer synthesis with inline particle size measurements for target DPs of 50, 150, 250, 350, 450, 550, and 650. A relationship developed between the targeted DP and resulting particle size was used in the second routine. This involved identifying the target DP required to synthesize nanoparticles of 50, 60, 70, and 80 nm and screening the polymerization kinetics using inline NMR. The final routine manufactured these nanoparticles of targeted size consecutively by running the reactor continuously at each corresponding target DP. The calculated flow rates for all reactions are given in Tables S1–S3, and a detailed outline of the steps involved in these experiments is given in Figure S3.

## 3 Results and Discussion

### Screening of PDMAm_150_-*b*-PDAAm_*n*_ Block Copolymer Nanoparticles with Inline Particle Size Measurement

**Figure 2:**
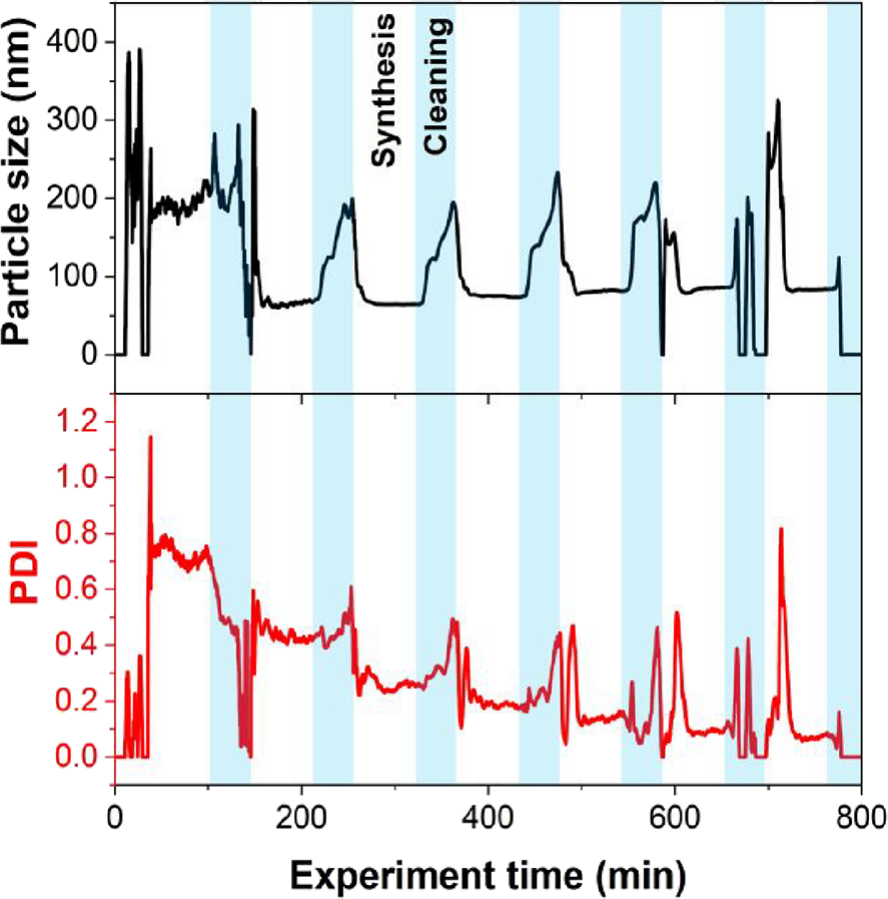
Time series (20-period moving average) of particle size (top) and polydispersity index (PDI) (bottom) measured during the automated screening experiment by inline SRDLS for seven PDMAm_150_-*b*-PDAAm_*n*_ block copolymers.

Two pairs of aqueous solutions A and B containing different concentrations of a preprepared PDMAm_150_ macro-CTA with initiator (A) and the DAAm monomer (B) were prepared as outlined in Section 2. The platform (Figure 1) comprising an automated tubular flow reactor with inline SRDLS and NMR was first programmed for establishing the relationship between the DP of PDAAm and the particle size (hydrodynamic diameter). The experimental structure is detailed in Figure S3. Briefly, this was achieved by simply entering minimum and maximum DP values to the interface (Figure S4) and then allowing the program to set the desired flow rates based on the material balance calculations (Equations S5–S8 in the Supporting Information) and run reactions with equally spaced values of DP. During each study, the SRDLS collected data every 10 s, which enabled real-time determination of size and polydispersity index (Figure 2) at the outlet in a noninvasive manner through a microflow cell. Within these data, the cleaning stages are also observed as peaks in particle size, which are likely a result of the particles swelling with methanol in the axially mixed region between the reaction and cleaning mixtures. Cleaning was included to remove any buildup of fouling from the reactor between each reaction.

**Figure 3:**
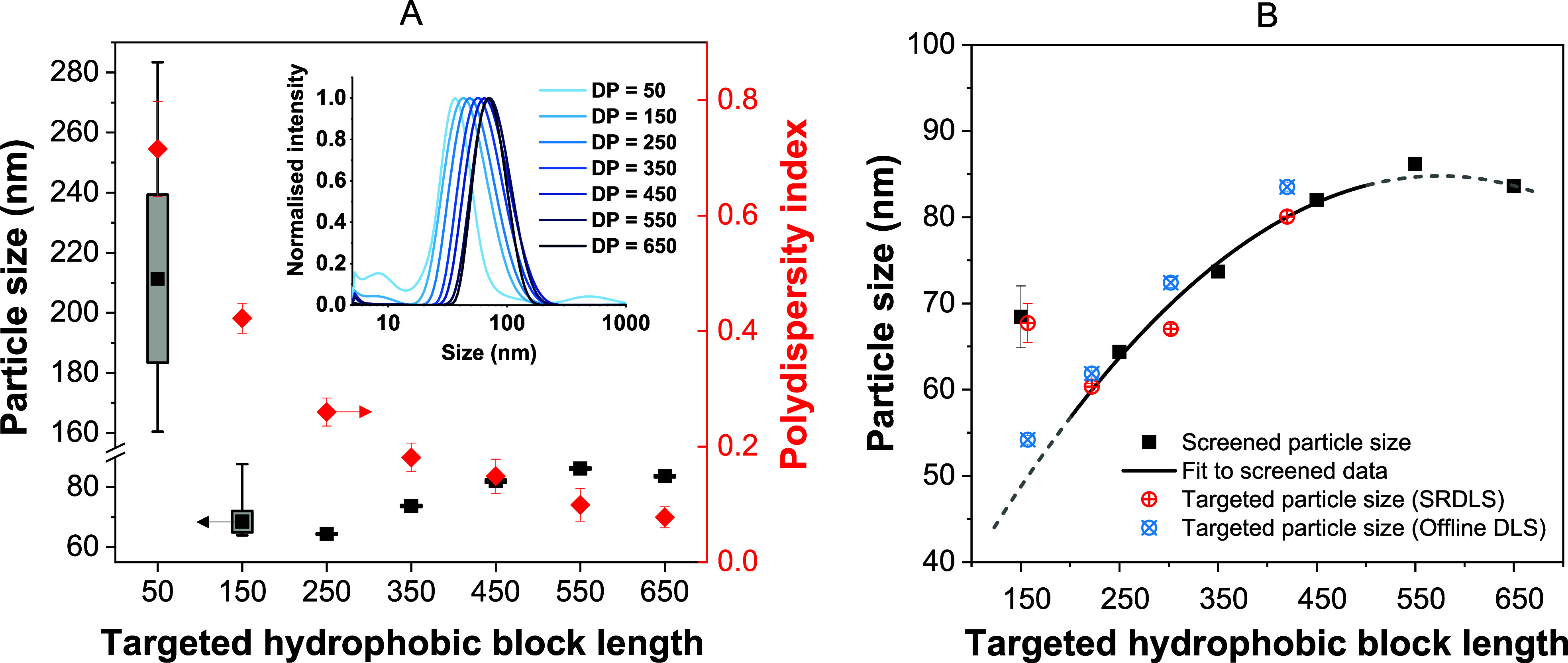
(A) Particle size summary with particle size distributions (inset) for seven PDMAm_150_-*b*-PDAAm_*n*_ block copolymers measured during steady-state sample collection by inline SRDLS. Boxes represent one standard deviation; whiskers show the min and max range. (B) Relationship between targeted hydrophobic block length and resulting particle size (black solid line) obtained from screening data (black squares). Dashed regions indicate areas of poorly defined particles (low DPs) or falling monomer conversion due to lower initiator concentrations (higher DPs). Target DPs of 50 and 150 were excluded from the fit due to particles being poorly defined. Measured particle size based on inline SRDLS (red symbols) and offline DLS (blue symbols) of block copolymer nanoparticles targeting particle sizes of 50, 60, 70, and 80 nm. Error bars representing one standard deviation of the measured size during the steady-state period are included for all samples.

After the conditions were set, it was necessary to wait for a steady-state operation before any sample could be collected. In this case, it was determined that allowing at least three reactor volumes to pass would be sufficient based on previous studies of the residence time distribution and polymer synthesis in tubular reactors.^49^ After this, the switching valve automatically diverted the outlet to a collection flask. This procedure enabled synthesis and collection of seven products (5 mL of each) in under 13 h, which were all characterized in situ by SRDLS. For target DPs between 250 and 550, a systematic increase in diameter was observed with increasing DP, indicating the growth of the block copolymer nanoparticles characteristic of PISA systems (Figure 3A).

In contrast, for the target DPs of 50 and (to a lesser extent) 150, the multimodality of the particle size distributions (from SRDLS) indicated multiple particle populations and a high polydispersity index (Figure 3A inset). This is likely a consequence of the PDAAm block being lower than the critical DP for formation of well-defined particles, which is between 150 and 250. For the longest targeted DP, an unexpectedly small particle size was recorded. In this case, the macro-CTA concentration is relatively low, and given the initiator concentration is based on this value (in this case 5:1 macro-CTA:initiator), the bulk initiator concentration is much lower than for the shorter DPs. Within the PFA coil, it has previously been observed that there is a requirement for a sacrificial initiator, which can quench oxygen that may be entering due to the tubing oxygen permeability.^51^ This is also shown in the work of Leibfarth and co-workers,^54^ who showed a dual-initiator strategy can aid in “polymerizing through oxygen” where a lower temperature initiator is employed as that sacrificial species. Here, at low bulk initiator concentrations, there is significantly slower polymerization initiation (with initial radicals acting as oxygen scavengers), accounting for the drop-off in conversion when the same residence time is employed. In principle, this could be mediated by increasing the residence time (with either a longer reactor or slower flow rates), but this would require further optimization beyond the scope of this work.

To effectively target values of particle size, it was necessary to consider only those formulations that resulted in well-defined particles for developing a relationship between the polymer structure and particle size. A minimum requirement for the quality of the particle size data obtained was therefore imposed. Where a standard deviation of particle size at steady-state exceeded a value of 1.0, it was deemed appropriate to consider that the particles prepared in these systems were not sufficiently defined and too irreproducible for the purposes of the proceeding studies. In the present case, it was deemed a second-order polynomial would fit the five remaining data points (Figure 3B) to target particle sizes in the next stage of the study. It is worth noting that the macro-CTA used in this study showed a reduced blocking efficiency (Figures S6–S8). This approach therefore enables the targeting of particle sizes regardless of the starting materials used. The presence of homopolymers may impact particle size; however, the approach presented can generate the desired particles irrespective of this.

### Kinetic Studies for the Synthesis of PDMAm_150_-*b*-PDAAm_*n*_ Block Copolymer Nanoparticles of Targeted Particle Size

Using the developed relationship, the flow rates required for nanoparticle sizes of 50, 60, 70, and 80 nm were programmed into the reactor. Programmed steady-state kinetic studies were conducted for each formulation to determine the residence time required for full conversion for the DAAm block (Figure S3, green boxes for methodology). For each sample, the reaction solution first bypassed the reactor to obtain an initial NMR spectrum before collecting samples after a sequence of six equally spaced residence times from 2 to 20 min. NMR spectra were collected after running at steady-state for three reactor volumes. Conversion was then determined by comparing integrals of the DAAm monomer vinyl region relative to those of the unreacted solution at each steady state.

**Figure 4:**
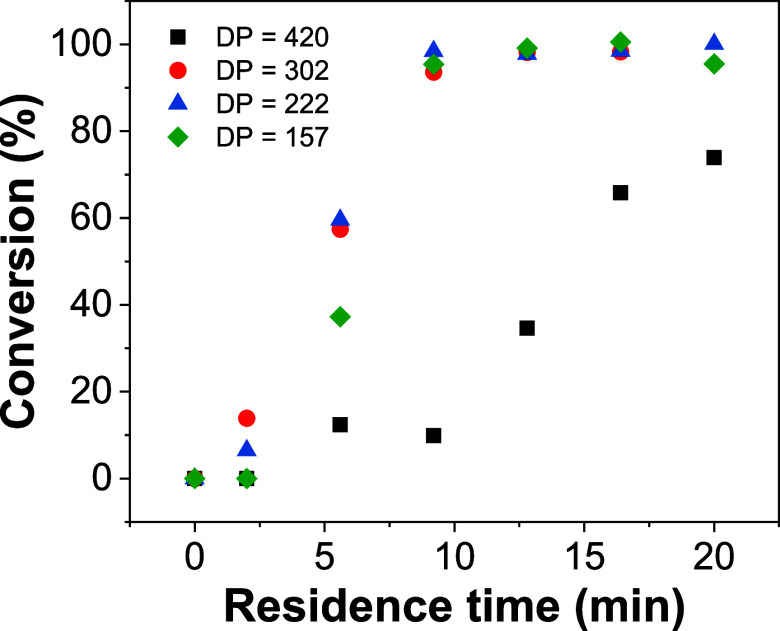
Kinetic studies of the formation of PDMAm_150_-*b*-PDAAm_*n*_ block copolymers at 75 °C for residence times ranging from 2 to 20 min for target DPs of 157, 222, 302, and 420. [CTA]:[initiator] = 5:1.

Near complete conversion (>95%) was attained after 9 min for the target DPs of 157 and 222 and 13 min for the target DP of 302 (Figure 4). As previously discussed, when targeting a DP of 420, a lower conversion of 73% was observed after 20 min due to the reduced initiator concentration, in conjunction with the oxygen permeability of the PFA tubular reactor. This explains the reduction in particle size observed for the highest target DP during the initial programmable screen and demonstrates the importance of being able to monitor the conversion during the process.

### Scale-up of PDMAm_150_-*b*-PDAAm_*n*_ Block Copolymer Nanoparticle Synthesis with Targeted Particle Size

**Figure 5:**
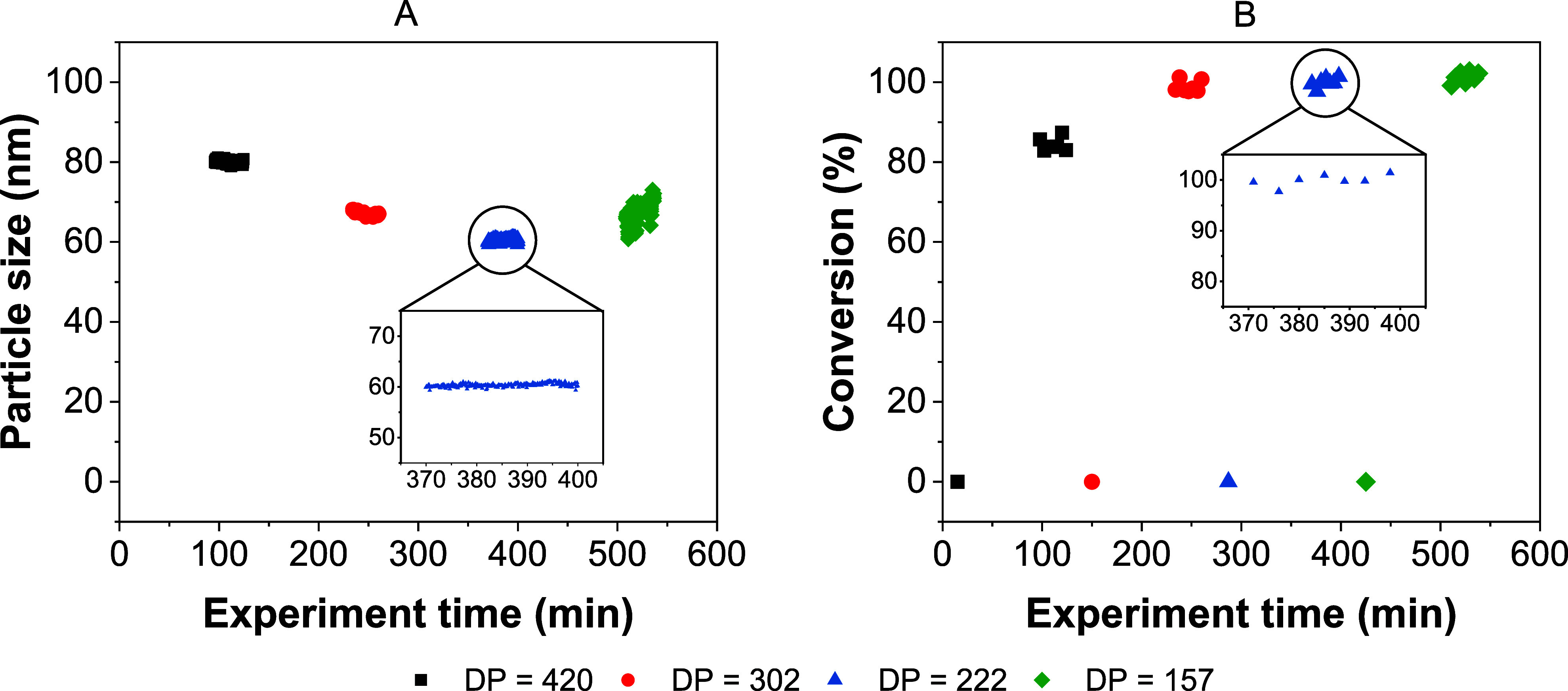
Measured particle size (A) and conversion (B) during the continuous manufacture of four PDMAm_150_-*b*-PDAAm_*n*_ block copolymers with targeted particles sizes of 80, 70, 60, and 50 nm at 75 °C for a 25 min residence time. [CTA]:[initiator] = 5:1. Inset graphs show the steady-state measurement periods for the target DP of 222. The data points at zero conversion represent the spectra obtained for the unconverted reaction mixture which bypassed the reactor before each synthesis.

Following the kinetic studies of the targeted products, the automated scale-up capability was evaluated. In each case, the target sizes were again programmed into the reactor, which then initiated the process of bringing the reactor to steady-state at the desired conditions before switching to collect sample for a period corresponding to the 10 mL sample volume (Figure S3, yellow boxes). During the sampling period, the particle size and conversion were continuously monitored to confirm the steady-state operation. The platform was programmed to synthesize 80 nm particles first, then reducing to 70, 60, and 50 nm. Time-resolved SRDLS data (Figure 5A) illustrates the in situ sizes of the samples collected, and the stable size measurements confirm steady-state operation during collection (full data in Figure S10). Online NMR (for spectra, see Figure S5) indicated near 100% monomer conversion (space time yield (STY) = 0.240 g/mL h) for samples with target sizes of 50–70 nm (Figure 5B), but this was reduced to 85% (STY = 0.204 g/mL h) for the 80 nm target sample as expected. In principle, conditions could be further optimized to bring the conversion of this sample closer to 100%. However, this would likely result in a deviation from the 80 nm target; conversion and size would need to be optimized simultaneously, which was beyond the scope of the present work. Data generated from the SRDLS indicated that the platform successfully targeted and automatically synthesized several samples of user-defined particle sizes (Figure 5A). The existence of a stable steady state is confirmed by the unchanging values of particle size and the conversion inset in Figure 5. The sample targeting a particle size of 50 nm appears unsteady according to the SRDLS data since the target DP of 157 is close to the region identified as generating poorly defined nanoparticles (Figure 3B). This region is seen to exist below a target DP of 150, but it likely extends slightly beyond that, accounting for the unsteady data obtained during the manufacture of this sample. The NMR data for this sample do however appear stable, confirming this as a feature of the particle size measurement.

Final particle sizes (60, 67, and 80 nm) were consistent with the targeted size (60, 70, and 80 nm, respectively) based on the relationship developed during initial screening (Figure 3B). Data from the synthesis targeting 50 nm (DP = 157) shows deviation from the expected smaller size; however, validation of this sample using offline DLS indicated the presence of well-defined 54 nm particles. We propose that this could be explained by one or a combination of reasons. First, the larger relative hydrophilic block at lower DPs is likely to reduce the driving force for particle formation as the chains are more soluble. Hence, the kinetics of self-assembly is much slower when the DP of the hydrophobic block is small. During inline measurement shortly after the reactor outlet, the self-assembly kinetics may be sufficiently slow that the instrument was making its measurement during this dynamic stage of particle formation. By the time these samples were characterized offline by DLS, the particles would have had sufficient time to become fully assembled resulting in a single particle population at a size more congruent with the relationship developed for the well-defined particles. This effect is more pronounced for the target DP of 50 than 150, with both samples resulting in a monomodal particle size distribution according to the offline measurement (Figure S12). This result suggests that SRDLS used in this way could offer new insights into the mechanism of particle nucleation during RAFT-PISA. Other factors that may contribute to the differences observed between SRDLS and offline DLS were also considered. For DPs at or around the critical DP for self-assembly, polymer chains are only weakly associated into loose aggregates.^55^ The shear forces associated with advective transport (wall shear of approximately 462 s^–1^, see SI) during inline analysis could be sufficient to disrupt this structure such that the chains become further dissociated and exhibit a larger hydrodynamic diameter in the SRDLS. A third cause may be that around this critical DP and due to the residence time distribution of the flow reactor (which is known to broaden molecular weight distributions^52,56^), there is likely to be a mixture of loosely formed aggregates and more well-defined nanoparticles. These larger aggregates will scatter more intensely in the SRDLS due to the increased wavelength of light used (1300 nm vs 633 nm for the offline DLS) causing a larger particle size measurement in samples containing both.

## 4 Conclusions

In summary, two inline analytical instruments work together with an automated flow reactor to enable the programmable synthesis of block copolymer nanoparticles at defined sizes and high conversion. Integration of SRDLS for noninvasive size monitoring and NMR for kinetic studies enabled a novel approach for rapid reaction screening to target size and determine reaction times, which directly informed continuous manufacturing. Furthermore, each product was automatically collected for further analysis, demonstrating an opportunity to rapidly synthesize and test a broad range of polymer products on demand using this platform. In principle, there is no reason that additional analytical instruments cannot be integrated into the platform to provide further opportunities (e.g., gel permeation chromatography). As such, this research presents powerful insight into the future operation of polymer chemistry laboratories.

## Supporting Information Available

Additional experimental details including photograph of reactor platform; material balance equations; an automated experimentation flowchart; user interface information; NMR spectra; gel-permeation chromatography data; a detailed description of SRDLS; DLS intensity distributions, including a comparison of SRDLS and offline DLS; and calculations of STY and wall shear rate (PDF)
