# Supplementary material for: Exploiting Online Spatially Resolved Dynamic Light Scattering and Flow-NMR for Automated Size Targeting of PISA-Synthesized Block Copolymer Nanoparticles
Source: ACS Polym Au. 2024 Nov 26;5(1):1–9. doi: 10.1021/acspolymersau.4c00074 (PMC11826489; doi:10.1021/acspolymersau.4c00074)
Supplement: Supplementary file 1 — lg4c00074_si_001.pdf [file lg4c00074_si_001.pdf]

Supporting information (SI) for:

Automated Size Targeting of PISA-Synthesized Block Copolymer Nanoparticles Using Inline Spatially-Resolved DLS and Flow NMR

Peter M. Pittaway,[a,‡] Kudakwashe E. Chingono,[a,‡] Stephen T. Knox,[a†] Elaine Martin,[a] Richard A. Bourne,[a] Olivier J. Cayre,[a] Nikil Kapur,[b] Jonathan Booth,[c] Robin Capomaccio,[c] Nicholas Pedge[c] and Nicholas J. Warren*[a†]

[a] School of Chemical and Process Engineering, University of Leeds, Woodhouse Lane, Leeds, LS2 9JT, UK.

[b] School of Mechanical Engineering, University of Leeds, Woodhouse Lane, Leeds, LS2 9JT, UK.

[c] Pharmaceutical Technology & Development, Operations, AstraZeneca, Silk Road Business Park, Macclesfield, SK10 2NA, UK

† Current address: School of Chemical, Materials and Biological Engineering, University of Sheffield, Sir Robert Hadfield Building, Mappin Street, Sheffield, S1 3JD, UK

‡These authors contributed equally.

Reactor platform photograph

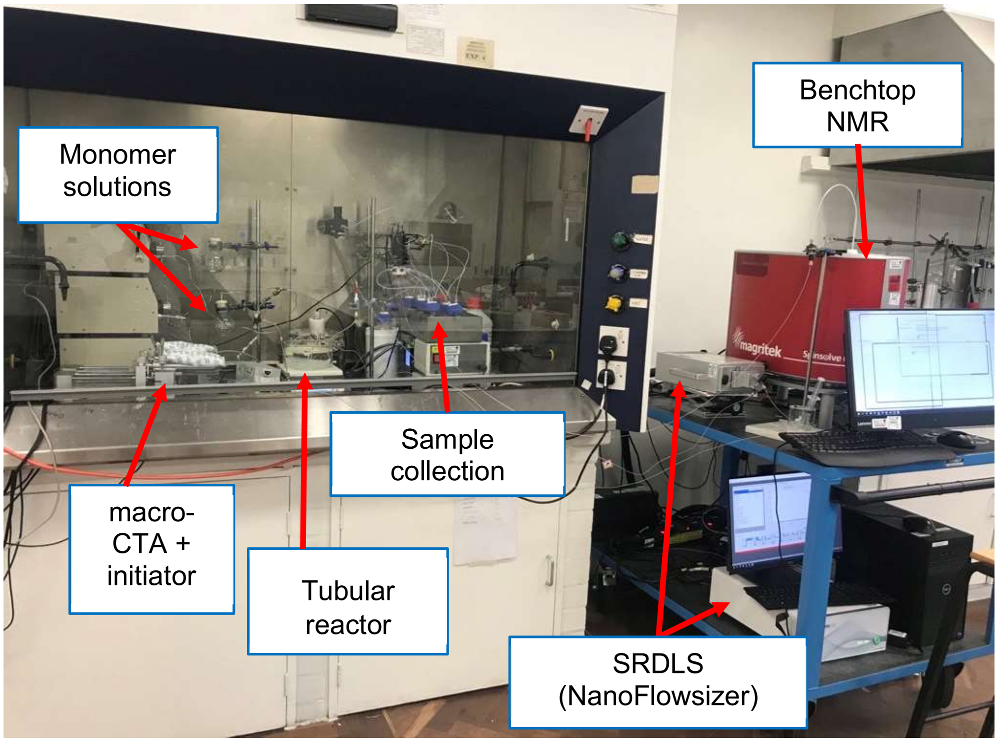

Figure S1. Photograph of the reactor platform for automated synthesis and characterisation of block-copolymer nanoparticles. Reagents are delivered from pumps on the left to the tubular reactor submerged in a temperature-controlled oil bath. The reactor outlet tubing flows into the SRDLS instrument and the benchtop NMR which is then directed to sample collection.

Material balance equations for calculation of relative flow rates

For a fixed product concentration and CTA : initiator ratio, the following material balances to calculate the required makeup of the four flasks (Figure S2) were required to carry out the automated experiments.

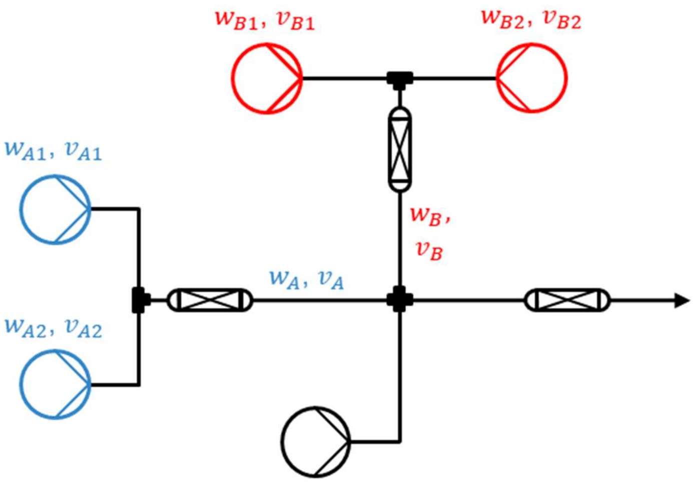

Figure S2. Schematic of reagent feeding 'front end' of the copolymer screening platform. Flasks A1 and A2 represent solutions containing macro-CTA and initiator, and Flasks B1 and B2 represent solutions of monomer.

The user first selects the final product concentration, w_f, and the range of copolymer block lengths ($(DP)_{max}$ to $(DP)_{min}$) to be explored before providing molecular weights of initiator, M_I, macro-CTA, M_{mCTA}, and monomer, M_m. In this way, the molar flow rate of each reagent can be controlled whilst keeping the final product concentration the same.

$$w_{A1} = \frac{2w_f\rho_f}{\rho_A(M_{mCTA} + M_m(DP)_{max})}\left(\frac{M_I}{R} + M_{mCTA}\right) \qquad (S.1)$$

$$w_{A2} = \frac{2w_f\rho_f}{\rho_A(M_{mCTA} + M_m(DP)_{min})}\left(\frac{M_I}{R} + M_{mCTA}\right) \qquad (S.2)$$

$$w_{B1} = \frac{2w_f M_m(DP)_{max}}{\rho_B(M_{mCTA} + M_m(DP)_{\mathrm{max}})} \qquad (S.3)$$

$$w_{B2} = \frac{2w_f M_m(DP)_{min}}{\rho_B(M_{mCTA} + M_m(DP)_{\mathrm{min}})} \qquad (S.4)$$

For any targeted degree of polymerisation, $(DP)_{target}$, the required solids fraction to be supplied by the combined flows of flasks A1 and A2, w_A, and by the combined flows of flasks B1 and B2, w_B, are calculated from Equations S.1 and S.3 by replacing $(DP)_{max}$ with $(DP)_{target}$. For a known reactor volume, V, and a defined residence time, τ, the required flow rates for the four pumps are calculated from the following. All densities were assumed to be 1 g/mL on the basis of the low concentrations in water, though the equations are constructed to facilitate systems which could deviate from this.

$$v_{A1} = \frac{\rho_A V(w_A - w_{A2})}{2\tau\rho_{A1}(w_{A1} - w_{A2})} \qquad (S.5)$$

$$v_{A2} = \frac{\rho_A V(w_A - w_{A1})}{2\tau\rho_{A2}(w_{A2} - w_{A1})} \qquad (S.6)$$

$$v_{B1} = \frac{\rho_B V(w_B - w_{B2})}{2\tau\rho_{B1}(w_{B1} - w_{B2})} \qquad (S.7)$$

$$v_{B2} = \frac{\rho_B V(w_B - w_{B1})}{2\tau\rho_{B2}(w_{B2} - w_{B1})} \qquad (S.8)$$

The calculated concentrations of the combined feeds (w_A and w_B) and flow rates of each pump are summarised in Tables S1, S2, and S3 for the initial screening, kinetics screening, and continuous manufacture experiments respectively.

Table S1. Calculated feed concentrations and pump flow rates for the synthesis of PDMAm$_{150}$-*b*-PDAAm$_n$ block copolymers in the initial screening experiment. [CTA] : [initiator] = 5 : 1, concentration = 10% w/w, residence time = 25 minutes.

Target DP	w_A	w_B	v_{A1}	v_{A2}	v_{B1}	v_{B2}
**50**	0.129	0.072	0.000	0.170	0.000	0.170
**150**	0.075	0.125	0.088	0.082	0.088	0.082
**250**	0.053	0.147	0.123	0.047	0.123	0.047
**350**	0.041	0.159	0.143	0.027	0.143	0.027
**450**	0.033	0.167	0.155	0.015	0.155	0.015
**550**	0.028	0.172	0.164	0.006	0.164	0.006
**650**	0.024	0.176	0.170	0.000	0.170	0.000

Table S2. Calculated feed concentrations and pump flow rates for the kinetic screening of $PDMAm_{150}$-*b*-$PDAAm_n$ block copolymer synthesis. [CTA] : [initiator] = 5 : 1, concentration = 10% w/w.

τ (min)		Target DP = 157	Target DP = 222	Target DP = 302	Target DP = 420
2.0	w_A	0.073	0.058	0.046	0.035
	w_B	0.127	0.143	0.154	0.165
	v_{A1}	1.137	1.447	1.686	1.902
	v_{A2}	0.988	0.678	0.439	0.223
	v_{B1}	1.137	1.447	1.686	1.902
	v_{B2}	0.988	0.678	0.439	0.223
5.6	w_A	0.073	0.058	0.046	0.035
	w_B	0.127	0.143	0.154	0.165
	v_{A1}	0.406	0.517	0.602	0.679
	v_{A2}	0.353	0.242	0.157	0.080
	v_{B1}	0.406	0.517	0.602	0.679
	v_{B2}	0.353	0.242	0.157	0.080
9.2	w_A	0.073	0.058	0.046	0.035
	w_B	0.127	0.143	0.154	0.165
	v_{A1}	0.247	0.314	0.367	0.414
	v_{A2}	0.215	0.147	0.095	0.048
	v_{B1}	0.247	0.314	0.367	0.414
	v_{B2}	0.215	0.147	0.095	0.048
12.8	w_A	0.073	0.058	0.046	0.035
	w_B	0.127	0.143	0.154	0.165
	v_{A1}	0.178	0.226	0.263	0.297
	v_{A2}	0.154	0.106	0.069	0.035
	v_{B1}	0.178	0.226	0.263	0.297
	v_{B2}	0.154	0.106	0.069	0.035
16.4	w_A	0.073	0.058	0.046	0.035
	w_B	0.127	0.143	0.154	0.165
	v_{A1}	0.139	0.176	0.206	0.232
	v_{A2}	0.120	0.083	0.054	0.027
	v_{B1}	0.139	0.176	0.206	0.232
	v_{B2}	0.120	0.083	0.054	0.027
20.0	w_A	0.073	0.058	0.046	0.035
	w_B	0.127	0.143	0.154	0.165
	v_{A1}	0.114	0.145	0.169	0.190
	v_{A2}	0.099	0.068	0.044	0.022
	v_{B1}	0.114	0.145	0.169	0.190
	v_{B2}	0.099	0.068	0.044	0.022

Table S3. Calculated feed concentrations and pump flow rates for the continuous manufacture of $PDMAm_{150}$-*b*-$PDAAm_n$ block copolymers. [CTA] : [initiator] = 5 : 1, concentration = 10% w/w, residence time = 25 minutes.

Target DP	w_A	w_B	v_{A1}	v_{A2}	v_{B1}	v_{B2}
**157**	0.073	0.127	0.091	0.079	0.091	0.079
**222**	0.058	0.143	0.116	0.054	0.116	0.054
**302**	0.046	0.154	0.135	0.035	0.135	0.035
**420**	0.035	0.165	0.152	0.018	0.152	0.018

Automated experimentation flowchart

The flowchart below (Figure S3) describes the operations involved for performing the automated exploration of the block copolymer synthesis. Colours represent the different stages of the experiment starting with the manual preparation phase (blue), the initial screen of DP versus particle size (red), the size targeting and kinetic screening (green), and the continuous manufacture of the targeted products (yellow). Whilst it would be entirely feasible to run each stage consecutively without user input beyond the preparation phase, for the purposes of collecting samples for offline validation, the three stages were performed independently with samples collection for each.

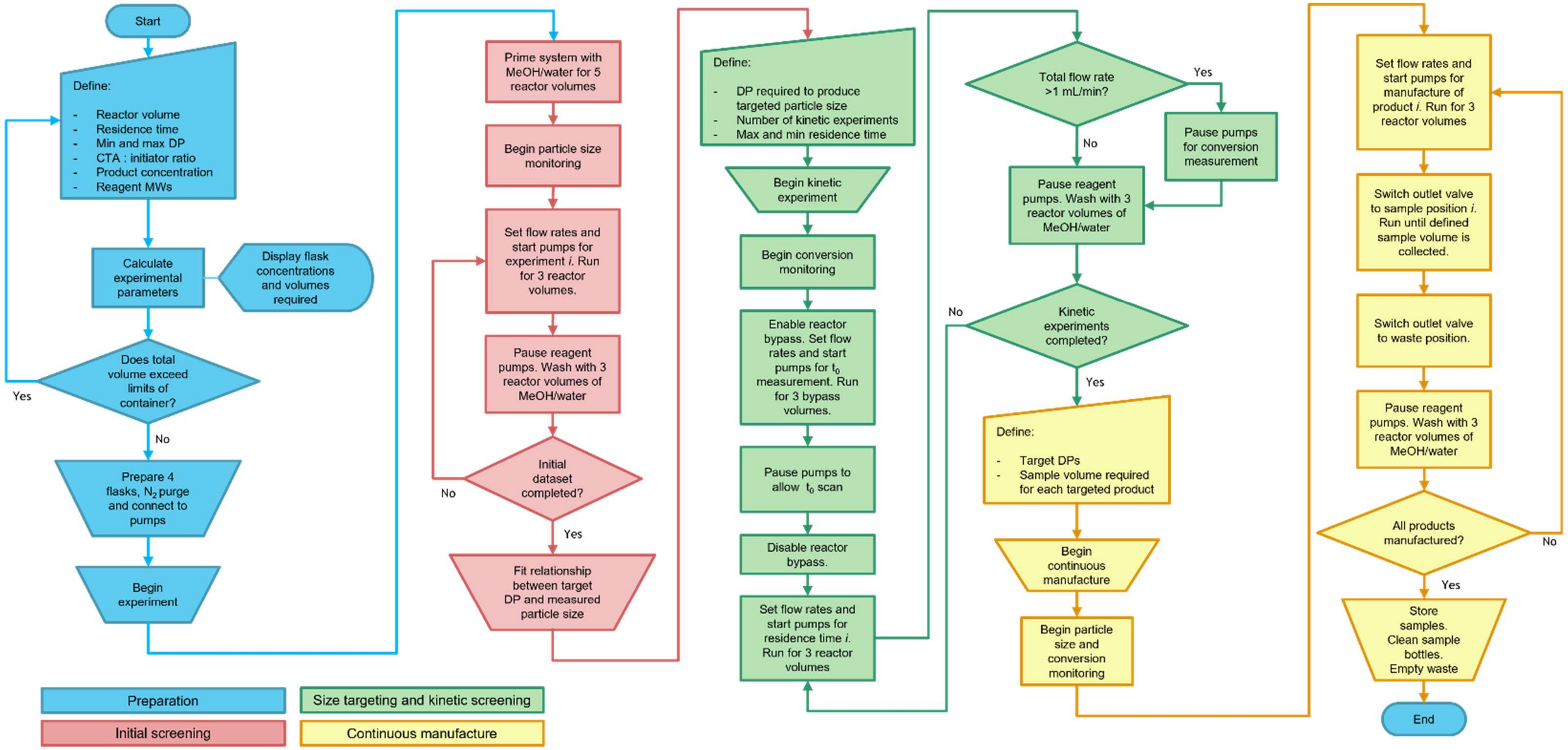

Figure S3. Flowchart representing the automated exploration of block copolymer nanoparticles. After the preparation phase (blue), the three separate modules can be used to screen particle size versus DP (red), evaluate the kinetics of producing targeted particle sizes (green), and continuously manufacture those targeted products (yellow).

The graphical user interface (GUI) made in Python using the PyQt5 library is shown below for defining the experimental conditions and controlling all pumps and valves.

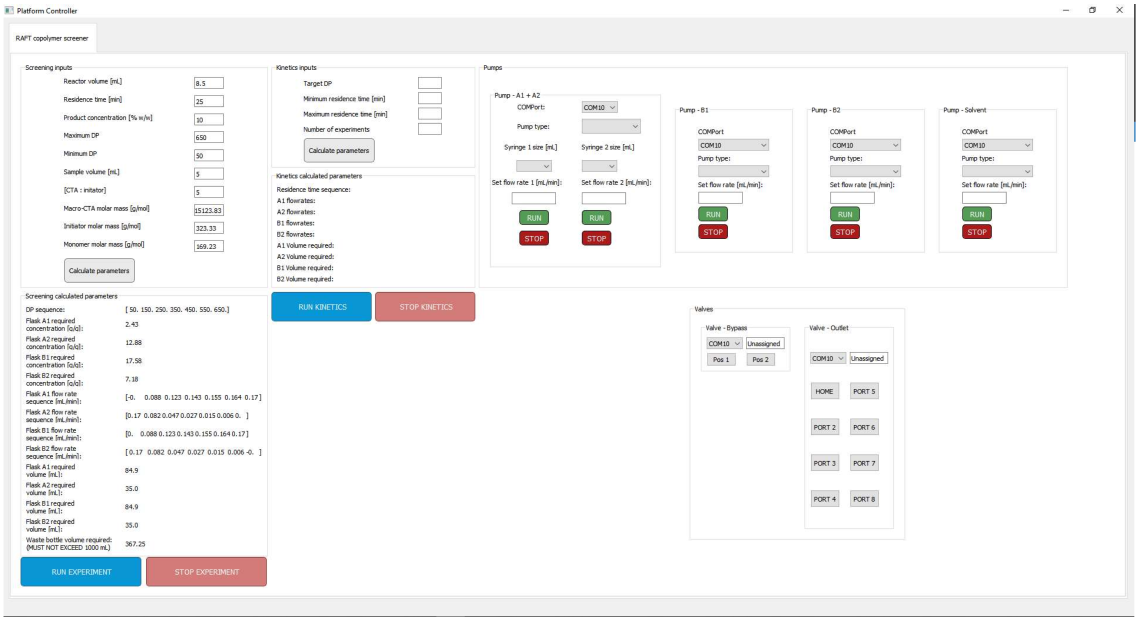

Figure S4. Graphical user interface for defining the automated experiments with example inputs. The continuous manufacture of targeted products was performed using the automated screening inputs with larger sample volumes.

Preliminary screening

An initial dataset was obtained by continuously monitoring the particle size during the sequential synthesis of 7 block copolymers with DPs ranging from 50 to 650 at intervals of 100. A cleaning step was programmed in between each reaction to wash the system with the MeOH/water mixture for at least three reactor volumes. Though it would be straight forward to run more than seven reactions at this stage, this number was selected so that each could be sampled using the 8-way collection system for offline validation of the inline particle size measurements.

Kinetic studies of targeted block copolymers

Based on the screening data, a relationship was obtained between the targeted DP and the final particle size such that pump flow rates could be calculated to then target specific particle sizes. The DP required to obtain four particle sizes of 80, 70, 60, and 50 nm was passed to the platform and kinetic studies of each product was performed automatically. For each kinetic study, the reactor was first bypassed to collect an NMR spectrum of the unreacted mixture, before the flow was diverted through the reactor at residence times of 2.0, 5.6, 9.2, 12.8, 16.4, and 20.0 minutes.

Continuous manufacture of targeted products

As a final step, the synthesis of the targeted products was scaled-up by continuously manufacturing 10 mL of each targeted product under steady-state operation. Of course, more than 10 mL could be collected simply by allowing more time to run.

Material characterisation

Nuclear magnetic resonance spectroscopy

A low-field (60 MHz) benchtop NMR spectrometer (Magritek) was placed inline downstream of the SRDLS for real-time conversion monitoring. Spectra were collected using a pre-saturation solvent suppression routine (1 s saturation pulse at 4.79 ppm of -70 dB, 7 µs excitation pulse, 5 kHz spectral width (32,768 points), and an acquisition time of 6.4 s (repetition time of 10 s, and number of scans = 2). Conversion was quantified by measuring the integrals in the alkenyl region (6.75-5.50 ppm) relative to its size in the unreacted solution. The unreacted solution was diverted automatically using a 6-way switching valve to bypass the reactor before switching back to start the reaction. Equation S.9 was used to calculate conversion, where the integral at 6.75-5.50 ppm for the unreacted solution was used as a measure of monomer concentration at time = 0 ($[M_0]$) and the same integral at time = t used as ($[M]$).

$$x_m = 1 - \frac{[M]}{[M]_0} \qquad (S.9)$$

Spectra obtained during the kinetic screen part of the automated routine are summarised in Figure S5.

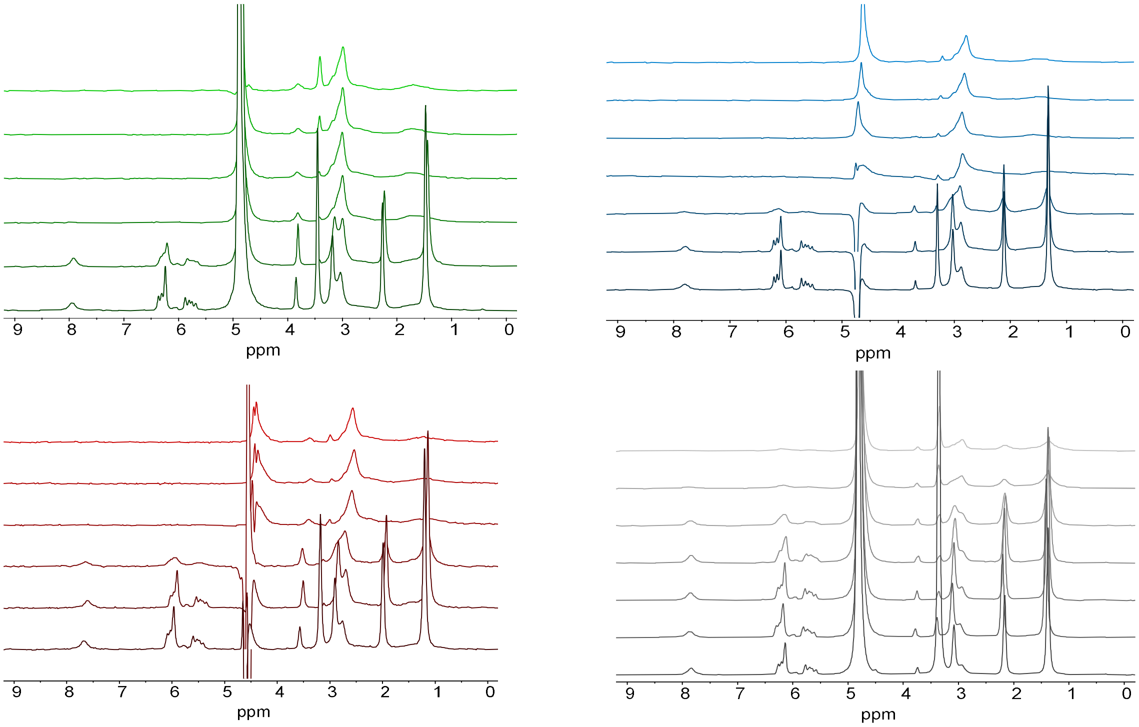

Figure S5. NMR spectra obtained during kinetic screening of four targeted block copolymer DPs of 157 (green), 222 (red), 302 (blue), and 420 (black) using an inline benchtop NMR.

Gel permeation chromatography

Molecular weight distributions were measured by gel permeation chromatography (GPC) for all samples obtained from the initial screening, kinetic screening, and continuous manufacture.

Offline GPC measurements were conducted with an Agilent 1260 Infinity system, fitted with two 5 µm Mixed-C columns (with guard column), an RI detector and a UV-Vis detector operating at 309 nm. DMF containing 0.1 % w/v LiBr was used as the eluent system at 1

ml/min, with the column oven and RI detector at 60 °C. A series of near-monodisperse PMMA standards were used (M_p: 800–2,200,000 g/mol) for calibration.

Chromatograms for the initial screen (Figure S6) indicate that the 25-minute residence time used was not sufficient for the higher DP targets to reach complete conversion as the value of M_n peaks at the target DP of 450. Additionally, the persistence of a second peak with M_n 15,000 g/mol suggests the presence of terminated chains in the PDMAm$_{150}$ macro-CTA, resulting in the larger than expected values of molecular weight for the copolymers prepared throughout the study.

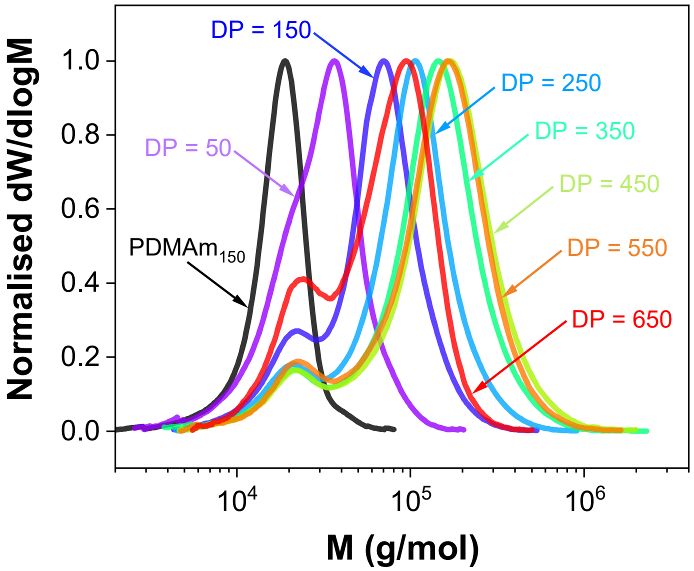

Figure S6. GPC chromatograms of PDMAm$_{150}$ macro-CTA and seven targeted PDMAm$_{150}$-*b*-PDAAm$_n$ block copolymers from the initial screen prepared at 75 °C for 25 minutes in water. [CTA] : [initiator] = 5 : 1.

Values of M_n and molar mass dispersity for these samples are summarised in Table S4.

Table S4. Summary of molar mass dispersity for the PDMAm$_{150}$ macro-CTA and the seven PDMAm$_{150}$-*b*-PDAAm$_n$ block copolymers from the initial screening experiment prepared at 75 °C, 10% w/w, for 25 minutes in water. [CTA] : [initiator] = 5 : 1.

Sample	M_n (g/mol)	*Đ*
**PDMAm$_{150}$**	15,800	1.18
**Target DP = 50**	25,200	1.36
**Target DP = 150**	43,400	1.63
**Target DP = 250**	58,600	1.84
**Target DP = 350**	71,900	2.13
**Target DP = 450**	81,300	2.23
**Target DP = 550**	77,000	2.16
**Target DP = 650**	45,400	1.70

GPC chromatograms of samples collected from the kinetic studies are presented in Figure S7 for targeted DPs of 222 and 302.

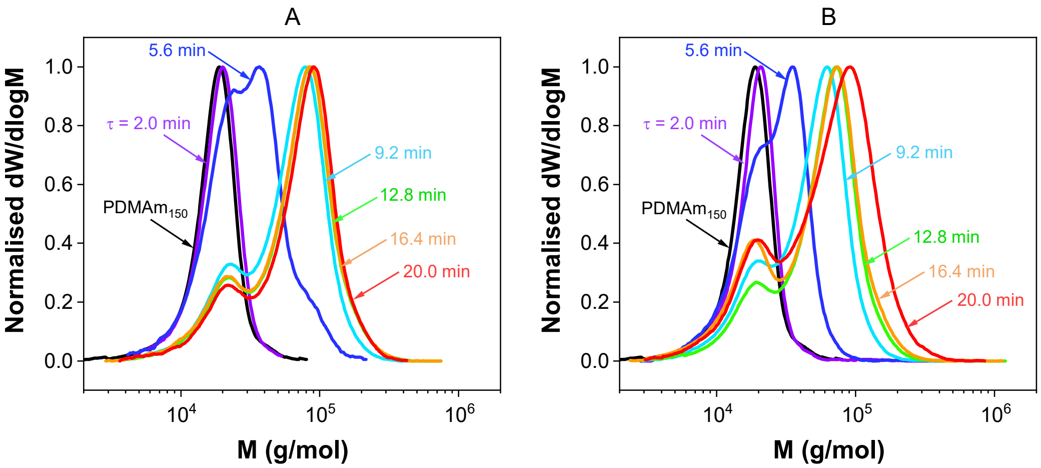

Figure S7. GPC chromatograms from kinetic screening of $PDMAm_{150}$-*b*-$PDAAm_n$ block copolymers targeting copolymer DPs of A) 222 and B) 302 corresponding to targeted particle sizes of 60 and 70 nm respectively. [CTA] : [initiator] = 5 : 1.

Molar mass dispersity of samples collected from the kinetic studies are summarised in Table S5.

Table S5. Summary of molar mass dispersity for $PDMAm_{150}$-*b*-$PDAAm_n$ block copolymers from the kinetic screening experiment prepared at 75 °C,10% w/w in water. [CTA] : [initiator] = 5 : 1.

**τ (min)**	**M_n (g/mol)**		***Đ***	
	Target DP = 222	Target DP = 302	Target DP = 222	Target DP = 302
**2.0**	16,300	16,800	1.36	1.19
**5.6**	23,700	21,700	1.48	1.33
**9.2**	40,300	33,200	1.65	1.57
**12.8**	45,600	39,100	1.73	1.62
**16.4**	43,400	32,100	1.78	1.90
**20.0**	42,400	37,900	1.75	2.01

MWDs of the four continuously manufactured products are shown in Figure S8 with target DPs of 157, 222, 302, and 420 corresponding to target particle sizes of 50, 60, 70, and 80 nm respectively.

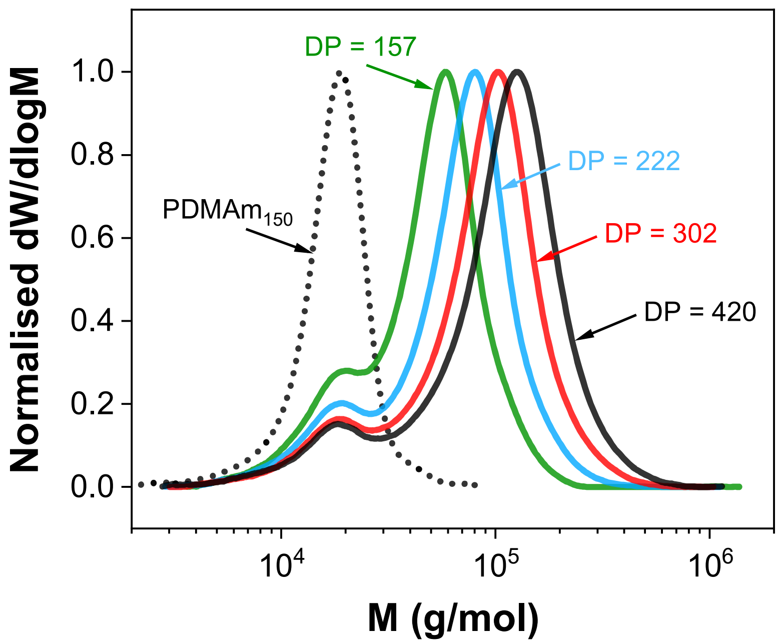

Figure S8. GPC chromatograms of four continuously manufactured PDMAm$_{150}$-*b*-PDAAm$_n$ block copolymers prepared at 75 °C for 25 minutes in water. DPs of 157, 222, 302, and 420 were targeted to produce particle sizes of 50, 60, 70, and 80 nm respectively. [CTA] : [initiator] = 5 : 1.

The persistent peak at ~15,000 g/mol for the block copolymer traces is attributed to terminated chains of the macro-CTA. Molar mass dispersities of these samples are summarised in Table S6.

Table S5. Summary of molar mass dispersity for the four targeted PDMAm$_{150}$-*b*-PDAAm$_n$ block copolymers prepared at 75 °C,10% w/w, for 25 minutes in water. [CTA] : [initiator] = 5 : 1.

Target DP	M_n (g/mol)	*Đ*
157	34,900	1.57
222	43,000	1.74
302	52,600	1.89
420	59,400	2.07

Particle sizing

Real time monitoring of particle size was achieved by installing the measurement head of the Spatially Resolved Dynamic Light Scattering (SRDLS) instrument, the NanoFlowSizer (InProcess LSP, Netherlands) downstream of the continuous flow reactor.[1] With this instrument, Fourier domain low coherence interferometry (FDLCI) combined with dynamic light scattering enabled dynamic size characterisation of polymeric nanoparticles in flow. In the proprietary measurement head, real-time sample temperature was measured by a probe and a low coherence light source (coherence length L_c ~1-10μm, centre wavelength of 1300 nm, data acquisition frequency of 48 kHz) illuminated the flowing sample in a micro flow cell (1mL volume, 3 mm depth, through an interferometer, light splitting optics and focussing lens. A 2.5 m optical fibre transmitted both incident and backscattered light to the spectrometer in the base unit. The Fourier domain analysis of the interference signals of the backscattered light with split incident light at specific path lengths (*z*) yielded scattered intensity profiles over time (*t*), I(z,t). Temporal intensity fluctuations due to diffusive Brownian motion and flow were thus obtained by measuring I(z,t) at microsecond time intervals. The instrument's proprietary flow correction algorithms decoupled flow motion from diffusive motion in the phase resolved signal to yield final autocorrelation functions at each pathlength in Figure S9.[1–3] A modified cumulants fitting method was used to obtain the cumulants Z-Average hydrodynamic diameter (d.nm) and a polydispersity index (PDI) for the expected monomodal distribution.[4] A MATLAB™ Client interfaced with the instrument using the Open Platform Communications Unified Protocol (OPCUA) for real-time monitoring, data collection and instrument control over a network connection.

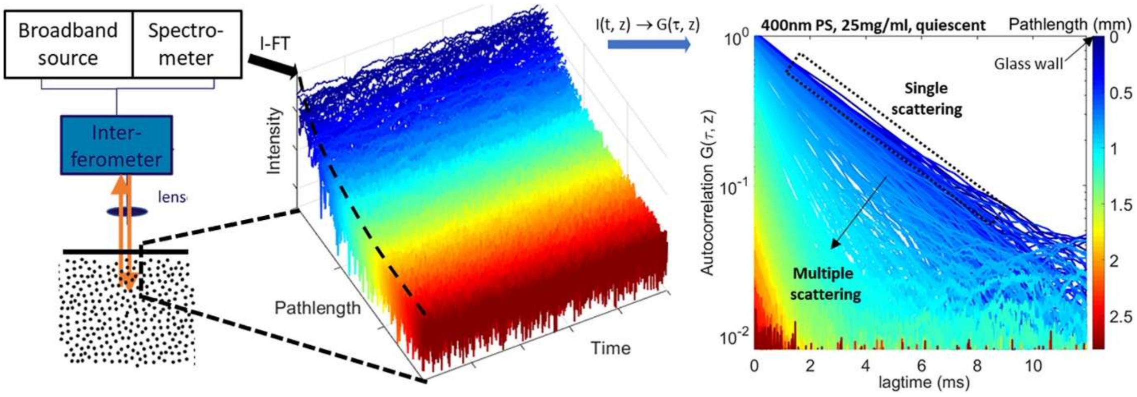

Figure S9. Schematic of the Fourier domain low coherence interferometry for Spatially resolved light scattering technique. Reproduced with permission from reference 1, © 2019 Elsevier B.V.

The time-series plot of the continuous manufacturing of block copolymers with targeted particle size is shown in Figure S10.

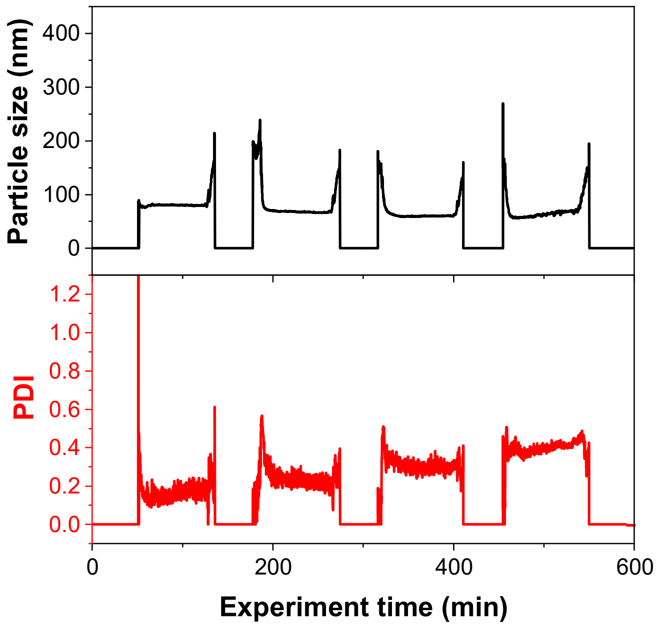

Figure S10. Time series of the four continuously manufactured $PDMAm_{150}$-*b*-$PDAAm_n$ block copolymers prepared at 75 °C for 25 minutes in water. From t = 0, target sizes of 80, 70, 60 and 50 nm with corresponding DPs of 420, 302, 222 and 157 sequentially produced without further input. [CTA] : [initiator] = 5 : 1. Stability of well-formed particles at higher DPs shown by longer stable steady-state sizes (top) and lower PDI relative to each other (bottom).

Dynamic light scattering

The size (hydrodynamic diameter, d.nm) of particles at steady state was validated offline by dynamic light scattering on a Malvern Zetasizer Advance (Malvern). Polystyrene cuvettes (path length, 10 mm) were used for the measurement with a 633 nm laser wavelength at a temperature of 25 °C and scattering angle of 173°. The cumulants fitting method was used to analyse the resulting correlation function to obtain the hydrodynamic diameter and PDI.[5] A regularised inverse Laplace method, CONTIN was used simultaneously to give intensity weighted particles size distributions (PSD).[6]

Intensity distributions from the static offline DLS measurements are shown in Figure S11 for (A) the initial screening experiment and (B) the continuous manufacture of targeted particle sizes.

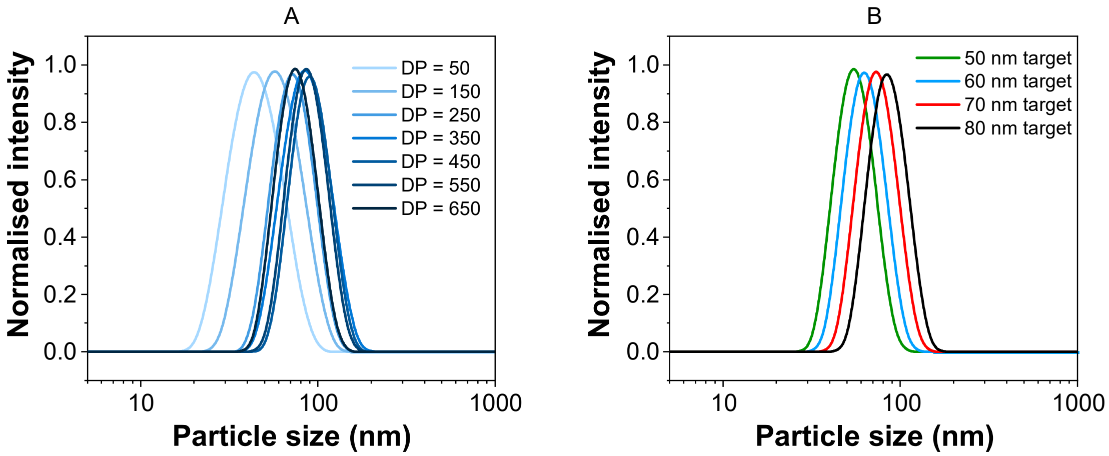

Figure S11. Particle size distributions of PDMAm$_{150}$-*b*-PDAAm$_n$ as measured by static offline DLS for A) seven samples of the initial screening experiment, and B) the four targeted particle sizes.

Size differences between SRDLS and offline DLS at smaller target DPs

At lower target DPs (50 and 150), measurements from the SRDLS indicated a larger Z-average size as compared to the offline DLS measurements, whilst also exhibiting a large PDI due to having multiple particle populations (Figure S12(A)). Because of the large PDI, these samples were not considered as part of the relationship developed for particle size targeting. However, when the samples were later characterised by offline DLS, the resulting sizes were smaller with lower PDIs (Figure S12(B)), which is more like what would be expected according to the relationship developed from the fitting of the larger well-defined particles at higher DPs (Figure 3(B) of the main manuscript).

Whilst the Z-average sizes reported suggest large differences for these samples with small DP, there exists a major particle population close to the final size observed in the offline DLS (Figure S12(A)). As the particles continue to assemble, it appears that the main population may shift to the higher value observed offline as the structures which comprise the larger and smaller peaks are incorporated into the main particle population with time (Figure S12(A)).

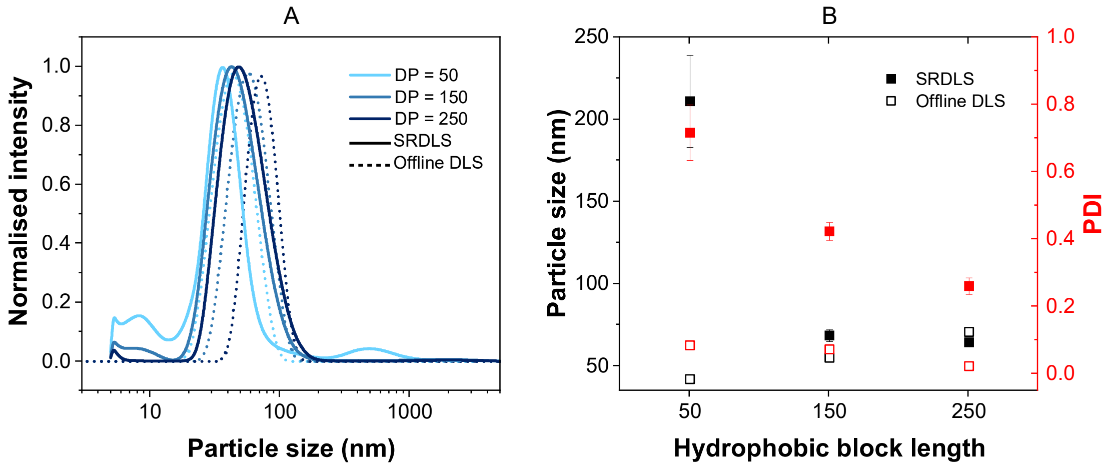

Figure S12. A) Particle size distributions of PDMA$_{150}$-*b*-PDAAm$_n$ block copolymers for target DPs of 50, 150, and 250 as measured by inline SRDLS and offline DLS. B) Summary values of Z-average particle size and PDI for the same samples.

Space time yield (STY) calculation

Space time yield (STY) was calculated by means of Equation S.10

$$\frac{m_{product}}{V \times \tau}$$

Where $m_{product}$ is the mass of product, V is the reactor volume and τ is the residence time.

Calculation of wall shear rate

A possible explanation for the increased particle size observed by the SRDLS at low DPs could be the break-up of loosely formed aggregates because of shear forces in the flowing material. For laminar flow in a tube of internal radius r, at a total flow rate Q, the shear rate at the tube wall, γ_w, can be calculated according to Equation S.11.

$$\gamma_w = \frac{4Q}{\pi r^3} \qquad (S.11)$$

For a flow rate of 0.34 mL/min in 1/16" PFA tubing (ID = 0.5 mm) as used in the manufacturing part of the present work, the wall shear rate is calculated as:

$$\gamma_w = \frac{4\left(0.34 \times \frac{10^6}{60}\right)}{\pi\left(0.5 \times \frac{10^{-3}}{2}\right)} = 462\ s^{-1}$$

References

(1) Besseling, R.; Damen, M.; Wijgergangs, J.; Hermes, M.; Wynia, G.; Gerich, A. New Unique PAT Method and Instrument for Real-Time Inline Size Characterization of Concentrated, Flowing Nanosuspensions. *Eur. J. Pharm. Sci.* **2019**, *133* (February), 205–213. https://doi.org/10.1016/j.ejps.2019.03.024.

(2) Lee, J.; Wu, W.; Jiang, J. Y.; Zhu, B.; Boas, D. A. Dynamic Light Scattering Optical Coherence Tomography. *Opt. Express* **2012**, *20* (20), 22262. https://doi.org/10.1364/oe.20.022262.

(3) Weiss, N.; Van Leeuwen, T. G.; Kalkman, J. Localized Measurement of Longitudinal and Transverse Flow Velocities in Colloidal Suspensions Using Optical Coherence Tomography. *Phys. Rev. E - Stat. Nonlinear, Soft Matter Phys.* **2013**, *88* (4), 1–7. https://doi.org/10.1103/PhysRevE.88.042312.

(4) Frisken, B. J. Revisiting the Method of Cumulants for Analysis of Dynamic Light Scattering Data. In *Optics InfoBase Conference Papers*; Optical Society of America, 2000; Vol. 40, p 95. https://doi.org/10.1364/ao.40.004087.

(5) Koppel, D. E. Analysis of Macromolecular Polydispersity in Intensity Correlation Spectroscopy: The Method of Cumulants. *J. Chem. Phys.* **1972**, *57* (11), 4814–4820. https://doi.org/10.1063/1.1678153.

(6) Provencher, S. W. CONTIN: A General Purpose Constrained Regularization Program for Inverting Noisy Linear Algebraic and Integral Equations. *Computer Physics Communications*. 1982, pp 229–242. https://doi.org/10.1016/0010-4655(82)90174-6.
